# Proton pump inhibitors with calcium acetate on serum phosphorus levels in hemodialysis patients

**DOI:** 10.22088/cjim.14.4.737

**Published:** 2023

**Authors:** Deniz Cekiç, Savaş Sipahi, Mehmet Emir Arman

**Affiliations:** 1 Department of Internal Medicine, Sakarya Research and Training Hospital, Adapazari, Sakarya, Turkey; 2 Department of Nephrology, Sakarya Research and Training Hospital, Adapazari, Sakarya, Turkey

**Keywords:** Renal failure, Hyperphosphatemia, PPI, Hemodialysis, Calcium acetate

## Abstract

**Background:**

The increase in serum phosphorus level is an independent risk factor for mortality in patients with chronic renal failure or undergoing dialysis due to end-stage renal disease. Proton pump inhibitors (PPI) are the general name given to agents used to suppress stomach acid. In this study, the clinical benefit of using PPIs in addition to drugs used for phosphorus control was investigated.

**Methods:**

153 patients with end-stage renal disease were included in the study. The data of the patients who had been on hemodialysis for at least 6 months and using calcium acetate for at least 1 month were recorded in the SPSS 21 program. The patients were analyzed in two groups according to whether they used PPI or not. Anamnesis, patient follow-up, laboratory, and treatment forms collected from hemodialysis centers were used.

**Results:**

Of the 153 patients in the study, 49% were males and the mean age was 65.11±11.23. The mean duration of patients on dialysis was 48.5 months. Hypertension was found to be the most common comorbidity with 75.8% prevalence among the patients. The mean phosphorus levels of the patients using calcium acetate together with PPI were found to be approximately 1.2 mg/dl lower (p= 0.000).

**Conclusion:**

It should be taken into account that the use of PPI together with calcium acetate, which is still common as a phosphorus binder in developing countries, can contribute to controlling phosphorus levels.

Electrolyte irregularity, a common problem encountered in chronic renal failure (CRF), is an independent risk factor for mortality, especially in end-stage kidney disease (ESKD) patients with uncontrolled hyperphosphatemia (1). Hypocalcemia and hyperphosphatemia are seen in chronic renal failure, both independently and in association with each other. It has been shown that increased phosphorus level causes renal osteodystrophy, pathological fractures in bones and calcification in vessels, and increases mortality and morbidity due to cardiovascular diseases (2). It is recommended to keep the blood phosphorus level between 3.5 mg/dl and 5.5 mg/dl to reduce the cardiovascular risk and increase the quality of life by reducing the risk of diuresis due to hyperphosphatemia (3).

 It was determined that mortality increased when calcium x phosphorus (CaxP) products were >70, and it was shown that mitral valve calcifications were present even when CaxP products were >55 in peritoneal dialysis patients (2, 4). In the guideline published by the Kidney Foundation–Kidney Disease Outcomes Quality Initiative (K/DOQI), it is recommended to keep the CaxP level as <55 (4).

Although the amount of phosphorus taken in the diet may not be sufficient in the control of phosphorus in patients with CRF, some agents should be used to reduce the phosphorus absorbed from the gastrointestinal tract in these patients. These agents are divided into aluminum-containing binders, calcium acetate-containing binders, calcium carbonate-containing binders and new generation phosphorus-binding agents. Although new agents have been developed for phosphorus control, calcium-based phosphorus-binding drugs are still used quite frequently (5).

Proton pump inhibitor (PPI) is the most effective treatment agent used for acid-related dyspeptic complaints for about 25 years. 

Although it is effective in the treatment of acid-related complaints and complications such as functional dyspepsia, Zollinger Ellison Syndrome, H. pylori, gastroesophageal reflux disease (GERD), and peptic ulcer by suppressing the acid secreted from the parietal cell in the stomach, there are new discussions regarding possible side effects. It is known that PPIs cause pathological bone fractures, neoplasia, acute interstitial nephritis and malabsorption of vitamin B12, calcium, iron, magnesium and phosphorus (6). In in vitro studies; It has been determined that phosphorus binding agents such as lantonium carbonate, calcium carbonate, sevalemer sodium are absorbed in a pH-dependent manner, and calcium acetate binds phosphorus better at high pH values (7).

## Methods

This study was a retrospective observational study and conducted with the permission of the ethics committee of Sakarya University Faculty of Medicine, dated 23/11/2018 and numbered 71522473/050.01.04/270 and between January 2018 and June 2018, 153 patients with the diagnosis of ESKD and between January 2018 and June 2018 in Sakarya/ Tukey provience. We investigated all 453 ESKD with dialysis treatment in Sakarya/ Turkey province and who received dialysis treatment for at least 6 months and used only calcium acetate and any kind of PPI for at least 1 month, participated. In our study, patients who were in the hemodialysis program and using phosphorus binders other than calcium acetate as a phosphorus binding agent, using calcium for vitamin D or other reasons were excluded. Patients using PPI were using omeprazole, pantoprazole, esomeprazole active ingredients at a dose of 1x1. The files of the patients were reviewed retrospectively and medication not interfered by authors. After exclusion, 153 of 453 patients; gender, age, duration of the hemodialysis program, the drugs used and the biochemical parameters of the patients were recorded in the SPSS 24 program. The mean value and standard deviation were used for quantitative values, whereas numbers and percentages were used to represent qualitative value. Shapiro-Wilk was used for normality test, and chi-square test was used to compare the qualitative values. Mann-Whitney U and T tests were used for quantitative values according to normality distribution. For statistical significance, p<0.05 was accepted. SPSS V20.0 (IBM SPSS Statistics for Windows, Version 20.0; Armonk, NY, USA) package program was used for statistical analysis.

## Results

Of the 153 patients in the study, 49% were males and the mean age was 65.11±11.23. The mean duration of patients on dialysis was 48.5 months. Hypertension was found to be the most common comorbidity with 75.8% prevalence among the patients (table-1). While all of the patients were using calcium acetate, when they were divided into two groups according to the use of PPI, the mean phosphorus levels of the patients using calcium acetate together with PPI were found to be approximately 1.2 mg/dl lower. (P=0.000) When both groups were compared according to calcium, CaxP products and albumin levels, no statistical difference was found. (Table 2). 

**Table 1:** Demographics characteristics of Patients

**Parameter**		
**Age (All patient)**		65.11±11.23
**Mean hemodialysis duration (months)**		48.5± 52.1
**Gender (Male)**		75 (%49.0)
**Comorbidites**	**Diabetes**	56 (%33.9)
	**Hypertension**	116 (%75.8)
	**Coronary artery disease**	53 (%34.6)

**Table 2:** Comparison of phosphorus levels by phosphorus binders

**Parametre**	**PPI + (n:109)**	**PPI – (n:44)**	**p**
**Phosphate**	5.12±0.92	6.25±0.93	0.000
**Calcium**	8.63±0.72	8.75±0.74	0.374
**CaxP**	47.2±13.23	54.04±8.99	0.20
**P** **TH**	582.9±1918.1	552.1±557	0.917
**Albumin**	3.76 (1.7-4.4)	3.9 (1.6-4.8)	0.657

PPI: Proton pump inhibitor, Cap: calcium x phosphorus, PTH: parathyroid hormone

## Discussion

Cardiovascular diseases are the primary cause of mortality in patients with end-stage renal disease (9). It has been shown that calcium phosphate crystals accumulate in the vascular endothelium in the early stages in hyperphosphatemia (6). In a study, it was determined that the relative mortality was 1.2 times higher in patients with phosphorus levels higher than 6.5 mg/dl when compared to those with a phosphorus level of 2.4-6.5 mg/dl. Furthermore, when parathyroid hormone (PTH) and calcium levels were analyzed, there was no mortality difference, but when CaxP products were examined, it was found that the relative mortality risk increased 1.34 times in patients with CaxP>72 (3). Moreover, a rise in mitral valve calcification was detected when CaxP products were >55 in peritoneal dialysis patients (5). In another study, peritoneal dialysis patients with mitral valve calcification were found to have a higher mortality (10). A low protein diet (0.4-0.8 gr/kg/day) is recommended to reduce phosphorus levels (11). PPIs are drugs that are frequently used for dyspeptic complaints, and it is thought that these drugs may cause pathological fractures, increased bacterial pneumonia risk, dementia, hypomagnesemia and myopathies in elderly patients (7). 

It is known that normal gastric pH is between 0.3-3.9 (11). Although there is conflicting data on the long-term use of PPIs, it has been found to increase gastric pH up to 6 (13). When phosphorus-binding molecules are classified as calcium-based and non-calcium-based, non-calcium-based phosphorus binders are known to be superior to calcium-based ones in controlling phosphorus level (14). When the use of phosphorus binding agents and PPI were investigated in the literature, it was found that serum phosphorus levels increased significantly when sevelamer carbonate, lanthanum and calcium carbonate were used together with PPI. It is also known that PPIs limit the phosphorus binding properties of lanthanum (15, 16). Calcium acetate is a calcium-based phosphorus-binding molecule, which is still widely used, especially in developing countries, due to its low cost (17). In an in vitro study, when the pH of the phosphorus binding molecules used in daily routine was investigated, it was found that lanthanum, sevelamer and calcium carbonate bind phosphorus better at pH: 3, whereas calcium acetate binds phosphorus best at pH: 6 (8). As for our study, in accordance with the reviewed literature, the mean phosphorus level was found to be approximately 1.1 mg/dl lower in patients using calcium acetate with PPI. It should be considered that the use of PPI together with calcium acetate, which is still common as a phosphorus binder in developing countries, can contribute to controlling phosphorus levels. But in this subject, randomized controlled study should be done.
